# Global Measurements of Brown Carbon and Estimated Direct Radiative Effects

**DOI:** 10.1029/2020GL088747

**Published:** 2020-07-01

**Authors:** Linghan Zeng, Aoxing Zhang, Yuhang Wang, Nicholas L. Wagner, Joseph M. Katich, Joshua P. Schwarz, Gregory P. Schill, Charles Brock, Karl D. Froyd, Daniel M. Murphy, Christina J. Williamson, Agnieszka Kupc, Eric Scheuer, Jack Dibb, Rodney J. Weber

**Affiliations:** ^1^ School of Earth and Atmospheric Sciences Georgia Institute of Technology Atlanta GA USA; ^2^ Cooperative Institute for Research in Environmental Sciences University of Colorado Boulder Boulder CO USA; ^3^ Chemical Sciences Laboratory National Oceanic and Atmospheric Administration Boulder CO USA; ^4^ Faculty of Physics University of Vienna Vienna Austria; ^5^ Institute for the Study of Earth, Oceans, and Space University of New Hampshire Durham NH USA

**Keywords:** aerosol, light absorption, brown carbon, radiation forcing, black carbon, biomass burning

## Abstract

Brown carbon (BrC) is an organic aerosol material that preferentially absorbs light of shorter wavelengths. Global‐scale radiative impacts of BrC have been difficult to assess due to the lack of BrC observational data. To address this, aerosol filters were continuously collected with near pole‐to‐pole latitudinal coverage over the Pacific and Atlantic basins in three seasons as part of the Atmospheric Tomography Mission. BrC chromophores in filter extracts were measured. We find that globally, BrC was highly spatially heterogeneous, mostly detected in air masses that had been transported from regions of extensive biomass burning. We calculate the average direct radiative effect due to BrC absorption accounted for approximately 7% to 48% of the top of the atmosphere clear‐sky instantaneous forcing by all absorbing carbonaceous aerosols in the remote atmosphere, indicating that BrC from biomass burning is an important component of the global radiative balance.

## Key Points

- Globally, biomass burning is a large source of light‐absorbing carbonaceous aerosol that directly affect the planetary radiation balance
- Transported over long distances, brown carbon is a significant component of these aerosols, but its contribution was highly variable
- Brown carbon contributed up to 48% of average clear‐sky instantaneous forcing by light absorption by carbonaceous aerosols

## 1 Introduction

Atmospheric aerosols affect the global radiative balance by scattering and absorbing radiation (Chýlek & Coakley, 1974). The main light‐absorbing component of aerosols is black carbon (BC) (Bond & Bergstrom, 2006; Horvath, 1993); however, some components of mineral dust (Sokolik & Toon, 1999) and organic aerosols (OAs) also absorb visible light. Organic chromophores in aerosol particles are the least well understood and are overall referred to as brown carbon (BrC) because they absorb most strongly in the ultraviolet (UV) and near‐visible wavelengths, resulting in a brownish or yellow appearance.

One known major source for BrC are products of incomplete combustion of fossil and biomass fuels (Chen & Bond, 2010; Desyaterik et al., 2013; Hecobian et al., 2010; Hoffer et al., 2006; Zhang et al., 2013). The complex molecular structures of organic chromophores are challenging to exactly determine, although nitroaromatic compounds have been identified in urban and biomass burning aerosols (Claeys et al., 2012; Lin et al., 2016). Other compounds, such as polycyclic aromatic hydrocarbon derivatives and polyphenols, may contribute to aerosol light absorption properties as well (Lin et al., 2016). Field observations of wildfires in California (Forrister et al., 2015), the Amazon (Wang et al., 2016), and Crete (Wong et al., 2019) have indicated that a large fraction of emitted BrC can be depleted over time by bleaching, with a half‐life varying between 9 and 24 hr. However, studies show a small fraction of emitted chromophores of high molecular weight resist bleaching. Low molecular weight chromophores that rapidly bleach would then mainly contribute to BrC absorption near sources, while high molecular weight chromophores with longer lifetimes could continue to contribute to light absorption in aged biomass burning plumes over large spatial scales (Di Lorenzo & Young, 2016; Wong et al., 2017).

Estimation of the global aerosol direct radiative effects (DREs) in past studies treated OA as wholly nonabsorbing (Bellouin et al., 2005; Haywood & Boucher, 2000), whereas a variety of recent studies have attempted to estimate the global radiative impact of BrC. These studies are limited by incomplete knowledge of BrC sources, sinks, evolution, and chemical composition‐driven optical properties, and there is little data to assess model predictions. They estimate that the global average top of atmosphere (TOA) BrC DRE, which is its instantaneous radiative impact on the Earth's energy balance (Heald et al., 2014), ranges from +0.04 to +0.57 W m^−2^, with BrC contributing from 20% to 40% of DRE from total carbonaceous absorbing aerosol (i.e., BC + BrC) (Feng et al., 2013; Jo et al., 2016; Lin et al., 2014; Saleh et al., 2015). These model simulations depend on parameterized BrC emissions, often based on the BC‐to‐OA ratio, or modified combustion efficiency (MCE) (Jo et al., 2016; Saleh et al., 2014). They also assumed an invariant (nonbleaching) BrC following emission. In contrast, Wang et al. (2018) included BrC bleaching utilizing a 1‐day photochemical lifetime and predicted a global BrC DRE of +0.048 W m^−2^ and a similar BrC contribution to DRE by all carbonaceous aerosol absorption (23%). Other modeling studies that included both bleaching and the added effect of BrC enhancement relative to BC with increasing altitude (Zhang et al., 2017) found that DRE due to upper troposphere BrC can largely offset BrC bleaching (Zhang et al., 2020). Model skill has often been assessed by comparison with BrC inferred from Aerosol Robotic Network (AERONET) data, but these data have substantial uncertainty itself (Schuster et al., 2016). Global‐scale data sets of measured BrC are needed for evaluation of model predictions and an assessment of its importance in the radiative balance. Here, the first estimates of BrC DRE and importance relative to BC, based on direct observational data over large spatial scales, is reported.

## 2 Method

### 2.1 The ATom Mission (Atmospheric Tomography Mission)

**Figure 1:**
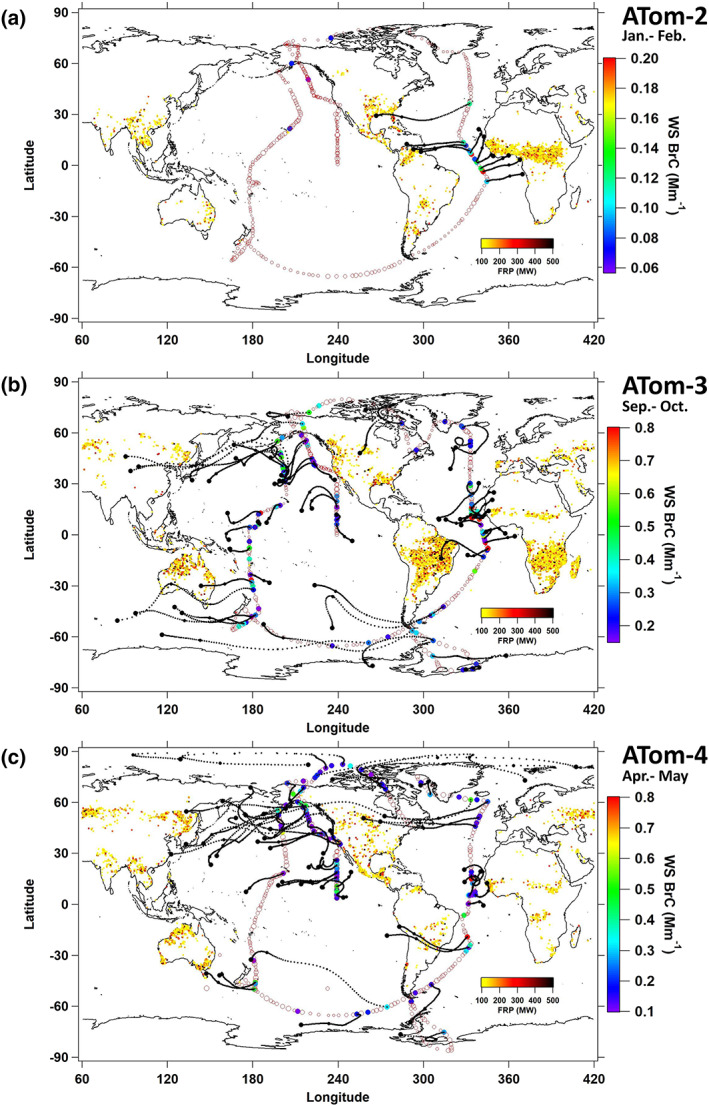
Water‐soluble (WS) BrC (absorption coefficient at 365 nm) global distribution measured in ATom‐2 (a), ATom‐3 (b), and ATom‐4 (c). Filled circles are colored by the magnitude of WS BrC for data above the LOD, and open circles represent data below the LOD, sized by relative magnitude. Fire dots with greater than 100‐MW fire radiative power (FRP) are colored by FRP magnitude in all plots. HYSPLIT air mass back trajectories are shown for up to 72 hr, where black dots indicate locations every 24 hr.

The National Aeronautics and Space Administration (NASA) DC‐8 aircraft conducted research flights nearly pole to pole along the central Pacific (north to south) and Atlantic (south to north) Oceans at altitudes systematically alternating from near surface (180 m) to ~13 km above sea level over four deployments, one in each season (see Table 1) (Prather et al., 2017). A map is shown in Figure 1. (BrC measurements were made in ATom‐2, ATom‐3, and ATom‐4 deployments.)

**Table 1:** ATom Data Summary

	ATom‐2	ATom‐3	ATom‐4
Deployment dates	26 Jan. to 21 Feb. 2017	8 Sept. to 27 Oct. 2017	24 Apr. to 21 May 2018
Number of filters analyzed	323	380	362
BrC LOD, Mm^−1^	0.05	0.15	0.10
% of filters above LOD	5.1	28.4	27.3
BrC Mean: Data below LOD set to 1/2 LOD, Mm^−1^	0.003	0.172	0.099s
BrC Median: No adjustment for below LOD (Median for only data above LOD), Mm^−1^	−0.001	0.066	0.042
	(0.098)	(0.276)	(0.172)
Water‐soluble BrC to Total BrC Ratio	N.A.	57%±17%	50%±16%
AAE mean: wavelength ranges from 300 to 500 nm	4.1	4.3	6.5
Number of FIRMS identified fire counts with FRP greater than 100 MW globally	13,905	33,070	18,408
BrC mean DRE, (BrC set to 0 for data below LOD), W m^−2^	+0.033	+0.29	+0.15
	(+0.01)	(+0.25)	(+0.11)
BC mean DRE, W m^−2^	+0.11	+0.31	+0.17
Scattering Mean DRE, W m^−2^	−8.07	−17.02	−8.99

*Note*. BrC absorption data are for only water‐soluble species. To estimate corresponding aerosol absorption coefficients, liquid absorption coefficients should be multiplied by a factor of 1.8 to 2 (see text). The direct radiative effect (DRE) was based on water‐soluble BrC multiplied by a factor of 4 to account for conversion of liquid measurement of chromophores to particle absorption and convert water‐soluble BrC to total BrC absorption (see WS BrC/Total BrC row above).

### 2.2 Filter Sampling, Extraction, and Analysis

The particle filter sampling system and offline analysis was identical to that used in two previous studies on the DC‐8, SEAC^4^RS, and DC3 and the method described in those publications (Forrister et al., 2015; Liu et al., 2015; Liu et al., 2014; Zhang et al., 2017). Particles with aerodynamic diameter less than nominally 4.1 μm (McNaughton et al., 2007) were collected onto Teflon filters over all altitudes at intervals typically <5 min at altitudes below 3 km and a maximum of 15 min for higher altitudes. For all three missions, 1,074 filters were collected and analyzed, including two or three blank filters per flight. Filters were extracted in water first and then methanol sequentially, and extracts were filtered by an in‐line syringe filter to filter out insoluble compounds like BC. Light absorption spectra of the extract were measured with a spectrophotometer utilizing a 2.5‐m‐long liquid waveguide. A schematic of the method is shown in supporting information Figure S1, along with a more detailed description of the method. Light absorption coefficient of chromophores in solution was calculated following Hecobian et al. (2010). In the following, the absorption coefficient averaged between 360 and 370 nm (avg. 365 nm) was used as a measure of particle BrC levels; water‐soluble BrC (WS BrC) was determined from Abs365nm for water extracts, while total BrC was the sum of Abs365nm for water and methanol extraction. BrC absorption Ångström exponents (AAE; Abs_λ_ = *C* · λ^−*AAE*^) were also determined from the measured spectra (see Figure S2 for example spectra). Note that all data presented here are at standard temperature and pressure (273 K and 1,013 mb); however, these are converted to ambient conditions for the radiative calculations discussed below.

Limit of detection (LOD) was determined by three times the standard deviation of field blank filters, combining all blanks from a given deployment (Table 1). (See also Figure S3 for frequency distribution of all WS BrC data relative to calculated LODs for each mission.) In the following analysis we focus on only WS BrC due to high blanks associated with methanol extractions. BrC measurement uncertainty was calculated by propagating the uncertainties from sample collection to data analysis and is estimated at 20%, where the uncertainty associated with subtracting the blank contributed 40% to 60% of this overall estimate.

The light absorption measured in this study are largely by individual chromophores (molecules) dissolved in solution, not the absorption of suspended particulate. This technique was used since it exclusively measures BrC optical properties (BC is not included), resulting in a highly sensitive approach required for this remote atmosphere study; however, when used for analysis of filters, the main limitation is blank variability, as noted above. Past studies, based on measured BrC aerosol size distributions and Mie theory, indicate that a multiplication factor of 1.8 to 2.1 (roughly 2 ± 0.2, or ±10%) can be used to estimate the light absorption by aerosol particles based on measurements of chromophores in the bulk liquid extracts (Liu et al., 2013; Shetty et al., 2019; Washenfelder et al., 2015; Zhang et al., 2017). We include this in the subsequent overall uncertainty analysis, which is discussed more below.

### 2.3 Other Measurements on the DC‐8, Back Trajectories, and Fire Events

Refractory black carbon (rBC, or just BC here) content of individual particles was measured with a single particle soot photometer (SP2). Integrated BC concentrations have been adjusted to account for accumulation‐mode BC outside of the SP2's detection size range (Schwarz et al., 2008), and in‐cloud measurements were removed based on cloud‐probe data. In the following analysis, solvent‐extracted BrC and SP2 BC are assumed to encompass all absorbing carbonaceous aerosols. Methanol has been shown to extract greater than 92% of BrC from laboratory‐generated smoke (Chen & Bond, 2010), but other forms of light‐absorbing aerosols from wild fires may not be included in BrC by solvent extraction (Shetty et al., 2019), nor SP2 BC measurements, which would lead to our under‐measuring carbonaceous aerosol absorption in this study (Adler et al., 2019). Aerosol scattering was derived from particle number size distributions for dry sizes between 2.7 nm to 4.8 μm in diameter, which were measured at 1 Hz using a suite of particle counters (Brock et al., 2019). The National Oceanic and Atmospheric Administration (NOAA) Particle Analysis by Laser Mass Spectrometry (PALMS) instrument was used to assess both the relative contributions and mass concentrations of biomass burning sources to the ambient aerosol that encompasses particles of sizes between 0.1 and 4.8 μm (Froyd et al., 2019; Schill et al., 2020).

Air mass 72‐hr back trajectories were computed using the Hybrid Single‐Particle Lagrangian Integrated Trajectory (HYSPLIT) analysis method (Rolph et al., 2017; Stein et al., 2015). Locations and fire radiative power (FRP) of large biomass burning regions for each ATom deployment were obtained from the Fire Information for Resource Management System (FIRMS, https://firms.modaps.eosdis.nasa.gov/map/). Fires of FRP greater than 100 MW are only included in the analysis. Air mass transport time from fire emissions to the point of aircraft sampling was estimated based on HYSPLIT back trajectories from the sampling location to the nearest FIRMS‐identified wildfire intersected by the trajectory. Type of fuel, or other variables that may affect emissions, were not considered. More details are provided in the supporting information.

### 2.4 Radiative Impact of BrC

Radiative transfer calculations were performed with the Santa Barbara DISORT Atmospheric Radiative Transfer (SBDART) model (Ricchiazzi et al., 1998) to compute the direct shortwave (0.25–4 μm) radiative effect at the top of the atmosphere (TOA). Accuracy of the model is discussed by Obregón et al. (2015) and more details are described in Zhang et al. (2017). Estimates of aerosol scattering from dry aerosol size distributions, and measurements of BC and BrC collected during aircraft vertical profiling were used in the calculations. In‐cloud data were excluded. Either for a complete ATom mission, or for a given geographical region, all vertical profile data were averaged (mean) and then used in the radiative calculation. The ambient aerosol scattering coefficient (b_scat) at multiple wavelengths was calculated with dry aerosol size distribution and measured temperature, pressure, and relative humidity (RH) using κ‐Köhler approximation for hygroscopic growth (Brock et al., 2016; Brock et al., 2019). Data were fitted with a power law (*b*_*scat*_ = *A* · λ^−*SAE*^, A is a constant, and SAE is the scattering Ångström exponent), which was then used with light scattering data to determine the aerosol scattering over all wavelengths in the radiative forcing calculation. The light absorption coefficient for BC (*b*_*BC*_) was calculated from the measured BC mass concentration using a mass absorption cross section (MAC) of 10.0 m^2^/g at 660 nm and AAE of 1 to compute absorption at other wavelengths. This is equivalent to a factor of 1.6 lensing effect due to BC coatings (i.e., for uncoated BC a MAC of 6.25 m^2^/g at 660 nm is typically used) (Zhang et al., 2017). If BC absorption is actually larger than this due to greater lensing effects, or AAEs > 1, we will overestimate the BrC contribution to radiative forcing. For BrC, measured absorption at 365 nm (Abs_365nm_) and an AAE value of 5 (the average of the measured WS BrC AAE, discussed below), was used to compute absorption at all wavelengths (*b*_*BrC*_).

The WS light absorption data were converted to an overall aerosol BrC absorption coefficient by the combination of two factors. First, the factor to convert WS BrC to total BrC in solution. Based on our data (Table 1), the ratio of WS BrC to total BrC for all ATom data is 53% ± 17%. Other studies have reported the WS BrC to total BrC ratio for aged aerosols to be in the range of 25% to 80% (Chen & Bond, 2010; Liu et al., 2015; Phillips & Smith, 2017; Satish & Rastogi, 2019; Shetty et al., 2019; Wong et al., 2017; Zhang et al., 2013). Here we assume the ratio is 0.5 with ±40% uncertainty, meaning the WS BrC is multiplied by 2 (±40% uncertainty) to estimate the contribution of all chromophores to BrC. We then convert the chromophores absorption to an aerosol light absorption coefficient. This factor depends on the particle size distribution of BrC, which, as discussed above is, estimated to be a factor of 2 ± 0.2 (10%), meaning the overall conversion factor is 4. More recent simultaneous measurements in smoke plumes of aerosol absorption with a photoacoustic instrument and the same BrC filter sampling system utilized here show an overall conversion ratio of 3.21 (*r* ^2^ = 0.84) for WS BrC to aerosol absorption at a wavelength of 405 nm, consistent with the factor of 4 here, considering uncertainly (see Figure S4). Including the uncertainty in BrC measurement of 20% (discussed above), we estimate the overall BrC aerosol light absorption coefficient determined by this method has an uncertainty of ±46%.

To parse out the various aerosol contributions to TOA radiative effects, we performed three SBDART runs to determine: (1) DRE due to only scattering, (2) DRE due to scattering and BC absorption, and (3) DRE due to scattering, BC absorption, and BrC absorption. We estimated the DRE of BC by subtracting (1) from (2), and the DRE of BrC by subtracting (2) from (3). More model details are provided in the supporting information.

## 3 Results and Discussion

### 3.1 Global Distribution of Fires and BrC

BrC measured in ATom‐2, ATom‐3, and ATom‐4 is shown in Figure 1, along with air mass back trajectories for those regions where WS BrC was above the LOD. Locations of burning are shown with indicated FRP, for fires with FRP > 100 MW. We find that WS BrC was very low over vast areas (also see Table 1); however, there were regions of significant WS BrC; these include the mid‐Atlantic Ocean, northern Pacific Ocean, and southern Pacific Ocean near islands in Oceania (Australia, New Zealand, etc.).

In the tropical or mid‐Atlantic region, enhanced levels of WS BrC were recorded in all three missions. FIRMS‐identified wildfires and back trajectories suggest that the BrC source for this region was either fires in South America or Africa. In ATom‐2 (January–February), most fires were in equatorial regions in northern South America and Africa, coinciding with the dry period for these regions (January–April). These measurements accounted for a majority of the observed BrC above LOD for the complete ATom‐2 mission. During ATom‐3 (September–October), the fires in South America were found further south, following the movement of dryer regions southward, dictated by the annual movement of the Inter‐Tropical Convergence Zone. Compared to ATom‐2, the wildfires were also more extensive in ATom‐3 in terms of both fire density and radiative power (FRP). In the last mission, ATom‐4 (April–May), the extent of fires in these regions decreased to the lowest levels relative to the ATom‐2 and ATom‐3 missions. Levels of BrC recorded in the mid‐Atlantic tracked these seasonal trends.

For the North Pacific Basin in Figure 1, WS BrC was observed in ATom‐3 and ATom‐4 and back trajectories indicated that the BrC was from northeastern China but occasionally from fires in western North America. Nearly no WS BrC was above the LOD in ATom‐2 in this region, which could be due to differences in emissions and transport with season. BrC from biofuels or other forms of incomplete combustion may also contribute but would not be evident from the FRP data. For the tropical mid‐Pacific Ocean (Figure 1), BrC above LOD was only observed in ATom‐3 and ATom‐4, possibly from scattered islands in the region, such as Hawaii. In the South Pacific, BrC was observed downwind of Indonesia, Australia, and New Zealand (Oceania), mainly during ATom‐3, suggesting it was also highly seasonal. For example, the Oceania region fire counts with FRP greater than 100 MW during ATom‐2 was 419, while there were 6,721 and 3,749 counts during ATom‐3 and ATom‐4, respectively. BrC was occasionally above LOD when sampling near or within polar regions (Antarctic and Arctic) during ATom‐3 and ATom‐4, where back trajectories show the air masses were mainly from high‐latitude regions, although it was difficult to locate specific sources for this region. BrC in polar regions may persist longer due to low sunlight limiting BrC photochemical bleaching.

As can be seen in Figure 1, the number of fire events identified from FIRMS varied significantly with region and season (i.e., ATom deployment). In general, trends of fire counts and levels of WS BrC were similar; highest fire counts were mainly seen in ATom‐3 and highest BrC levels were generally recorded in that mission (Table 1). However, significant scatter in this relationship can be expected since the aircraft did not necessarily sample plumes from all fires identified by FIRMS and there are uncertainties in both WS BrC and fire events identified by Moderate Resolution Imaging Spectroradiometer (MODIS) (Schroeder et al., 2008). Overall, we conclude that biomass burning appears to be the predominant source for BrC in the remote atmosphere since most regions of recorded BrC could be traced to a burning region. (We also found regions where measured BrC < LOD did not intercepted burning regions, see Figure S5). Given that the smoke plumes were transported over great distances (>10,000 km), some portion of the fire‐emitted BrC persisted for at least 3 days, the limit of our back trajectory analysis, consistent with laboratory studies that high molecular weight BrC species resist photobleaching (Di Lorenzo & Young, 2016; Wong et al., 2017).

### 3.2 BrC Correlation With BC

Correlations provide further evidence that the BrC was associated with mainly biomass burning. Biomass burning emits BC and BrC, although there are differences in emissions rates depending on fuel and burning temperature, and how these species may be altered with atmospheric age. More BrC is emitted per fuel burned in smoldering compared to flaming fires (Chakrabarty et al., 2016), whereas more BC is emitted in flaming than smoldering (Echalar et al., 1995). Some fraction of BrC will bleach over time, whereas BC is chemically stable, and only undergoes removal from the air with an estimated lifetime of about <5 to 10 days globally (Cooke & Wilson, 1996; Koch et al., 2009; Lund et al., 2018). Also, there is evidence that BrC is lofted to higher altitudes by convection more efficiently than BC (Zhang et al., 2017) (which was also observed in ATom as an increasing ratio of BrC to BC with increasing altitude, Figure S9); thus, some scatter between BrC and BC is expected even if both are emitted from wild fires in a given region. The Pearson correlation (r) between BrC and BC was 0.86, 0.75, and 0.53 for Atom‐2, Atom‐3, and Atom‐4, respectively (for scatter plots, see supporting information Figure S6, also see Table S1). Despite high correlations, there is significant variability at lower levels, suggesting that BC cannot solely be used to infer BrC optical effects. Data with moderate to low WS BrC, but very low BC, were mostly observed at higher altitudes (>9 km), possibly due to differences in advection of these species through clouds (Zhang et al., 2017), whereas periods (filter samples) that contained moderate to low BC but very low WS BrC were mainly found in the mid‐Atlantic Ocean region. Causes may be different burning conditions (smoldering/flaming) and processing during transport.

Comparing among separate ATom missions, the highest correlation between BrC and BC was found in ATom‐2 (0.86); the correlation was weaker in ATom‐3 (0.75) and lowest in ATom‐4 (0.53). A similar, although somewhat stronger correlation trend, was found for BrC versus estimated biomass burning potassium (K^+^_BB_) and between K^+^_BB_ versus BC (see Table S1 and supporting information discussion for calculation of K^+^_BB_). The trend was also seen in BrC and the PALMS estimate of biomass burning particle mass (see Figure S6). A possible explanation is BrC observed in ATom‐2 was mainly from two concentrated regions of burning (see Figure 1a), whereas in ATom‐3 and 4, data were from fires located in differing geographic regions. Thus, although the total impact of fires may be higher for ATom‐3 and Atom‐4, the characteristics of the emissions and effects during transport might be broader and more complex, which weakened the correlations.

### 3.3 DRE of BrC Aerosol

Light absorption over the full spectral wavelength range is necessary to simulate the radiative impact of BrC aerosol. As noted in section 2, we use a constant BrC AAE of 5, the mean for all missions. For the three ATom missions, AAE values ranged from 2.5 to 8.6 (10th and 90th percentiles) and the mean AAE was similar for ATom‐2 and ATom‐3 but higher for ATom‐4 (Table 1). No geographical dependence for AAE was observed, but higher AAE values were always found at high altitude or near the surface. The cause for variability in AAEs is not clear but adds uncertainty to model predictions of radiative effect, which we include in the overall estimated uncertainty.

**Figure 2:**
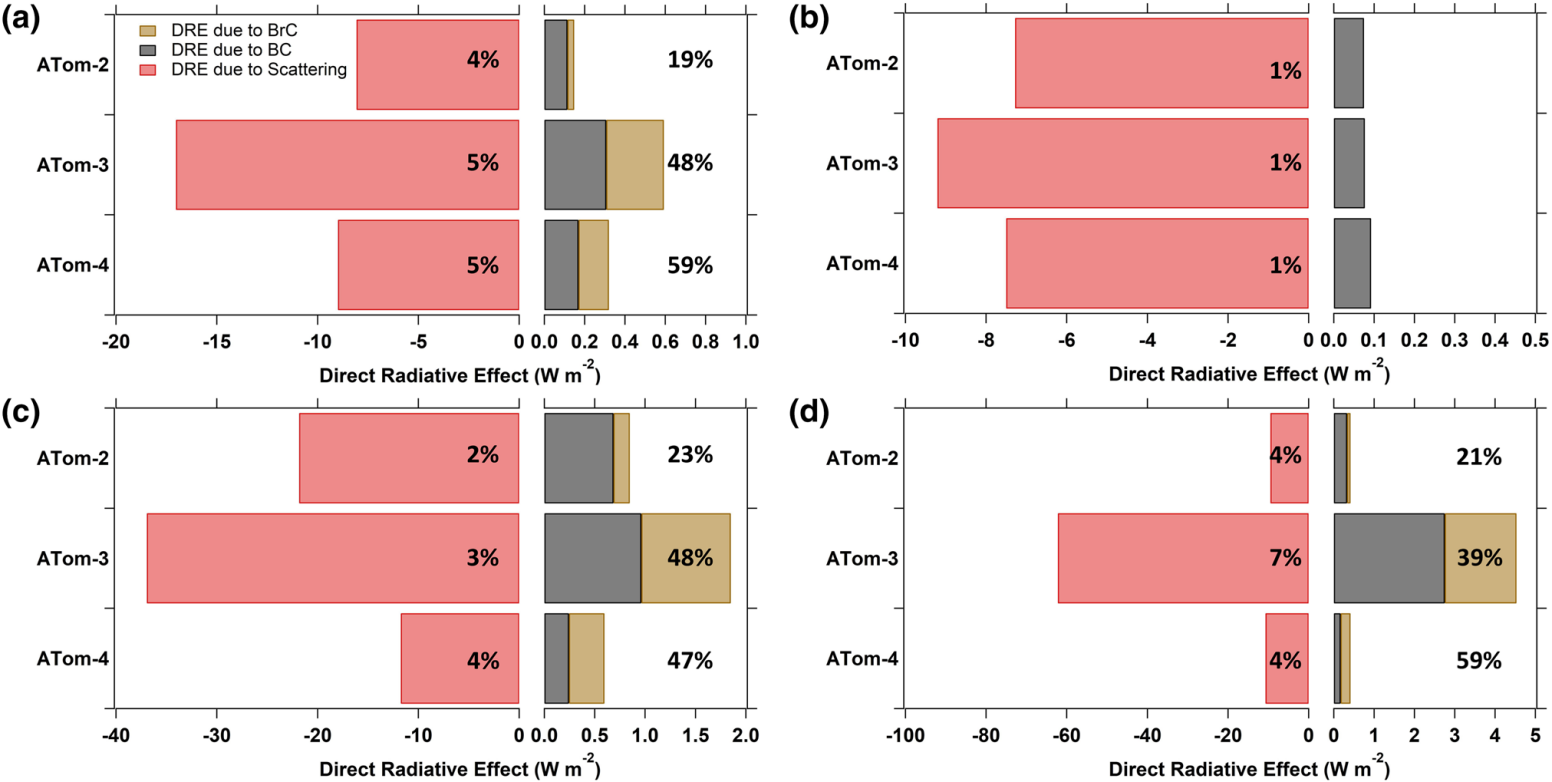
Instantaneous clear‐sky DRE at the top of atmosphere (TOA) computed with ATom data (a) for the average of each ATom mission when BrC data are above LOD, (b) average of each ATom mission when BrC data are below LOD, (c) global average of each ATom mission for all data with BrC < LOD set to 1/2 LOD, and (d) just for data in the mid‐Atlantic (see Figure 1). The percentages shown in the scattering bar are the fraction of DRE due to carbonaceous aerosol absorption relative to scattering, Abs (BC + BrC)/Scat., and the percentages shown to the right of the bar are the fraction of DRE due to BrC of all carbonaceous absorbing species, Abs (BrC)/Abs (BC + BrC). Note the scale changes at the point zero since aerosol scattering dominates TOA DRE in remote regions.

A summary of the radiative calculations is shown in Figure 2, where we compare averages for each ATom mission for different groups of data. (DREs of each aerosol component for various latitude ranges can be found in Table S2). Figure 2a shows the DRE for scattering, and BC and BrC absorption, for data in which the measured WS BrC was above the LOD. For just these data, BrC accounted for 19% to 59% of the carbonaceous aerosol absorption instantaneous forcing and carbonaceous aerosol absorption DRE offset total light scattering DRE by ~5%. These are periods (BrC > LOD) of sampling in plumes of fairly strong biomass burning influence, as confirmed by the PALMS tracer analysis; for the three ATom missions when BrC > LOD the median contribution of biomass burning to aerosol mass was 30% and median aerosol mass from biomass burning was 0.24 μg m^−3^. This contrasts with periods when BrC < LOD shown in Figure 2b, where the magnitudes of the TOA DRE was much smaller for scattering and absorption (contrast scales in Figures 2a and 2b). Based on the PALMS data, for these periods only 8% of the aerosol mass was from biomass burning and the concentration median was 0.03 μg m^−3^ (see bar and whisker plot in supporting information Figure S7). BC concentration followed a similar trend, BC was substantially higher when BrC > LOD (i.e., periods of smoke sampling), especially in ATom‐2 (Figure S8).

The average DRE for each mission was also calculated. Figure 2c shows the mean instantaneous DRE at TOA that includes all data, and where BrC < LOD was set to 1/2 LOD. The ratio of averaged DRE due to BrC was 44% of the averaged total light absorption by carbonaceous aerosols among three ATom missions. The mission average was 38% when BrC was set equal to 0 for BrC < LOD, (not plotted). The mean results are similar to those periods of BrC > LOD (Figure 2a) since the mean is dominated by the higher magnitude values. Figure 2d gives the DRE results for smoke detected in just the midtropical Atlantic, and Table S2 summarizes results from other latitude ranges. These data show that the BrC contribution can be substantial, but with significant variation, ranging from 21% to 59% of the total carbonaceous absorption DRE for the three ATom missions.

The fraction of carbonaceous aerosol DRE due to BrC for these data is similar to or surpasses the high end of the range reported by other studies (Feng et al., 2013; Jo et al., 2016; Lin et al., 2015; Saleh et al., 2014; Wang et al., 2016; Zhang et al., 2020). A possible reason is the models are truly global averages, whereas these are data only from where the aircraft sampled. Another possible reason is the sensitivity of DRE to the vertical distribution of BrC and BC, which most modeling studies may not correctly simulate. Throughout this study, BC was mainly found from near the surface to midaltitudes, whereas BrC was observed to decrease less slowly with altitude compared to BC, resulting in an increasing of BrC/BC with altitude (Figure S9), as has been seen in continental aerosols (Liu et al., 2014).

The approach used to investigate BrC based on dissolving aerosol in a solvent and measuring the molecular chromophores exclusive of BC generally has higher sensitivity than instrumentation that measures aerosol light absorption without altering the particle, such as the multiwavelength photoacoustic measurement. Even so, the majority of samples in the remote atmosphere were below detection limit using the solvent method. However, measuring dissolved chromophores and then estimating aerosol optical effects introduces uncertainty, as discussed in section 2. Sensitivity tests indicate that there is nearly a 1:1 correspondence between the change in BrC absorption coefficient and its DRE, implying the uncertainty in BrC TOA DRE is roughly ±45%, similar to the overall BrC absorption coefficient uncertainty at 365 nm. The use of a constant BrC AAE of 5, based on the average of all ATom data, also adds uncertainty to the DRE; the mean DRE due to BrC increases by about 10% for a BrC AAE of 3 instead of 5 and decreases by about 30% for an AAE of 7. Combining these uncertainties, we estimate the overall uncertainty in BrC DRE is roughly 50%. Assuming the uncertainty of DRE due to BC is significantly smaller, the fraction of BrC DRE to total carbonaceous DRE is estimated to be 7% to 23% for ATom‐2, 45% to 48% for ATom‐3, and 39% to 47% for ATom‐4 (range for setting BrC below LOD to 0 or 1/2 LOD) with ±24% uncertainty.

In summary, the smoldering combustion of wildfires is known to be a significant source of BrC. We find on a global scale, based on the regions measured during ATom‐2, ATom‐3, and ATom‐4 deployments (January/February, September/October, and April/May), that measurable amounts of BrC were associated with tracers of smoke such as BC, potassium, and PALMS single particle composition. Such smoke contained variable amounts of BrC, which was often detected great distances from the burning regions (greater than 10,000 km), persisting for more than 3 days following emissions. This BrC made a significant contribution to the overall absorption by carbonaceous aerosols and the top of atmospheric DRE; however, the spatial distribution of the BrC forcing was highly heterogeneous.

## Supporting information

Supporting Information S1

## Data Availability

The ATom data are available as described in Wofsy et al. (2018) and may also be accessed online (https://doi.org/10.3334/ORNLDAAC/1581). More detailed data (BrC and WSOC raw data) can be provided by contacting the corresponding author.
