# Supplementary material for: Global Measurements of Brown Carbon and Estimated Direct Radiative Effects
Source: Geophys Res Lett. 2020 Jul 1;47(13):e2020GL088747. doi: 10.1029/2020GL088747 (PMC7380307; doi:10.1029/2020GL088747)
Supplement: Supplementary file 1 — Supporting Information S1 [file GRL-47-e2020GL088747-s001.docx]

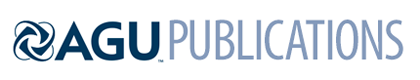


*Geophysical Research Letters*

Supporting Information for

Global Measurements of Brown Carbon and Estimated Direct Radiative Effects

Linghan Zeng^1^, Aoxing Zhang^1^, Yuhang Wang^1^, Nicholas L. Wagner^2,3^, Joseph M. Katich^2,3^, Joshua P. Schwarz^3^, Gregory P. Schill^2,3^, Charles Brock^3^, Karl D. Froyd^2,3^, Daniel M. Murphy^3^, Christina J Williamson^2,3^, Agnieszka Kupc^3,4^, Eric Scheuer^5^, Jack Dibb^5^, Rodney J. Weber^1^

^1^School of Earth and Atmospheric Sciences, Georgia Institute of Technology, Atlanta, GA 30332, USA

^2^Cooperative Institute for Research in Environmental Sciences, University of Colorado, Boulder, CO 80309, USA

^3^Chemical Sciences Laboratory, National Oceanic and Atmospheric Administration, Boulder, CO 80305, USA

^4^Faculty of Physics, University of Vienna, Vienna, Austria

^5^Institute for the Study of Earth, Oceans, and Space, University of New Hampshire, Durham, NH 03824, USA

**Contents of this file**

Text S1 to S4

Figures S1 to S9

Tables S1 to S2

**Introduction**

The following Supporting Information describes the analytical methods and data analyses in more detail. Additional figures and tables providing more details than those of the main article are also included below.

**S1. Methods Details**

Ambient particles were collected on a filter sampling system and then analyzed offline identical to that used in our previous studies. Based on earlier characterization studies, it was estimated that the inlet and transport system allowed sampling of particles with aerodynamic diameter less than nominally 4.1 μm [*McNaughton et al.*, 2007]. Particles were collected onto 1-μm pore size, 90 mm diameter, Teflon filters (MilliporeSigma, Burlington, MA), which were maintained at low temperatures (typically less than 0 °C) by refrigerating following each flight and during shipment to the Georgia Institute of Technology (Atlanta, GA, USA) in coolers with blue ice. 15 mL opaque polypropylene tubes (Argos Technologies, Vernon Hills, IL) were used in ATom-2 and 15 mL glass centrifuge tubes with Teflon lined caps (DWK Life Sciences, Rockwood, TN) were used for ATom-3 and ATom-4. The glass vials were baked at 500 °C for 24 hours to remove organic residue. Figure S1 illustrates the analytical system for the filter analyses.

Filters were first extracted in 15 mL of deionized water (DI water, >18.2 MΩ·cm) in its storage vial by 30 min sonication (FS60H Ultrasonic Cleaner, Fisher Scientific, Hampton, NH). For the analysis, the sample was injected via a programmable syringe pump (Kloehn Inc., Las Vegas, NV) through a pre-cleaned (flushed with extraction solvent) polypropylene syringe filter of 0.45 μm pore size (Tisch Scientific, North Bend, OH) and then into a 2.5 m long (total internal volume with 625 μL) liquid waveguide capillary cell (LWCC-3250, World Precision Instruments, Sarasota, FL). After the waveguide, the liquid sample was drawn into a total organic carbon analyzer (TOC analyzer, Sievers 900 Series, GE Analytical Instruments, Boulder, CO) to measure WSOC [*Hecobian et al.*, 2010]. (WSOC is not discussed in this paper). The LWCC was coupled to a dual deuterium and tungsten halogen light source (DH-mini Light Source, Ocean Optics, Dunedin, FL) and absorption spectrometer (USB4000 Miniature Fiber Optic Spectrometer, Ocean Optics, Dunedin, FL) via fiber optic cables. The spectrometer provided wavelength-resolved absorption spectra from 200 nm to 850 nm. These spectra were recorded by data acquisition software, SpectraSuite, every two seconds and were saved three times for each filter, which were then averaged to determine the final absorption spectra. After water extraction, filters and extraction vials were drained and passively dried for roughly 3-6 hours at room temperature. Then, the filters were extracted again using 15 ml of methanol by 30 minutes of sonication following the same method used for the water extracts (i.e., sample filtered and passed through LWCC), but without TOC analysis. Only light spectra were analyzed since the use of organic solvent prohibited the extract carbon mass quantification.

Light absorption coefficient of chromophores in solution ($\mathrm{Abs}_{\lambda}$, units m^-1^) was calculated using Eq (S1) [*Hecobian et al.*, 2010].

$\mathrm{Abs}_{\lambda}=\frac{V_{\mathrm{liquid}}\times\log_{10}\frac{I_{\lambda,0}}{I_{\lambda}}}{V_{\mathrm{air}}\times l}\times\ln(10),$ (S1)

where $I_{\lambda,0}$/$I_{\lambda}$ is the ratio of the measured intensity of incident to transmitted light through the waveguide at a given wavelength, $V_{\mathrm{liquid}}$ is the solvent volume used in the extraction, $V_{\mathrm{air}}$ is the sampling air volume passed through the filter and $l$ is the waveguide optical path length (2.5 m). Exclusive measurements of chromophores require subtracting out any absorbance by the solvent, buildup of contamination in the LWCC, or contribution of BC (if any). Daily, absorption reference spectra were produced by injecting pure DI water or methanol, depending on which extraction solvent was used. Then, during the subsequent sample analysis, prior to each filter analysis, pure solvent was injected into the analyzing system to monitor if a LWCC cleaning procedure was necessary. A routine cleaning procedure was done before and after daily experiments by injecting pure solvent into the LWCC. If necessary, but seldom for this study, 5 mL of 0.6N HCl solution was used to thoroughly clean the LWCC, and then at least 50 mL of DI water was flushed through the LWCC. Ideally, the result is that the absorption spectrum would be near zero at all wavelengths for pure solvent in the LWCC.

The light absorption coefficient determined by Eq(S1) at 700 nm ($\mathrm{Abs}_{700nm}$) was subtracted from measured $\mathrm{Abs}_{\lambda}$ (all wavelengths) to account for any baseline drift or any insoluble components (e.g., BC) that passed the liquid flow system and syringe filter, under the assumption that there is no BrC absorption at 700 nm. For ATom, $\mathrm{Abs}_{700nm}$ was typically very small meaning that few interferences from insoluble particles were present in the LWCC and baseline drift was minimal; in the worst case, strong smoke plumes, $\mathrm{Abs}_{700nm}$ was less than 20% of $\mathrm{Abs}_{365nm}$.

Methanol-soluble BrC (MS BrC) data were unreliable for all ATom missions due to high blanks. ATom-2 high blanks were thought to be due to the use of plastic extraction vials. Glass vials were than used for ATom-3 and 4. After this change, MS BrC still showed little contrast to the blank filter signal, and so in the following analysis we focus on only water-soluble BrC (WS BrC).

The data used in the following analysis are available in the ORNL DAAC ATom data archive [*Wofsy et al.*, 2018]. In these datasets, absorption coefficient data were averaged every 20 nm between 300 nm and 700 nm. The absorption coefficient at 365 nm, which is used here to represents the absorption by BrC, was determined by averaging from 360 nm to 370 nm (averaged to minimize noise). All data discussed below and uploaded in the data achieve were blank corrected by subtracting the average of the filter blank for each deployment and reported at standard temperature and pressure (273K & 1013 mb). It is important to note that the absorption measured in this study, discussed in this paper (unless otherwise indicated), and reported in the data achieve is the absorption by individual chromophores (molecules) dissolved in solution, not the absorption of suspended aerosols. Past studies, based on measured BrC aerosol size distributions and Mie theory, indicate that a multiplication factor of 1.8 to 2 can be used to estimate the light absorption by aerosol particles based on measurements of chromophores in the bulk liquid extracts [*Liu et al.*, 2013; *Y Zhang et al.*, 2017].

**S2. Back Trajectories and Fire Events**

Airmass back trajectories were computed using the Hybrid Single-Particle Lagrangian Integrated Trajectory (HYSPLIT) analysis method were calculated starting from the aircraft GPS location at the middle of the filter collection interval and traced back for up to 72 hours. Locations and fire radiative power (FRP) of large biomass burning regions for each ATom deployment were obtained from the Fire Information for Resource Management System (FIRMS). Although each mission lasted for approximately one month, no significant variation in regions strongly associated with FIRMS-derived fire events was observed throughout a given mission. The MODIS FRP retrieval, which was performed using the *Wooster et al.* [2005] approach, was used as a proxy for biomass burning emission rates.

**S3. Radiative Transfer Model**

For each altitude layer in the model, the particle single scattering albedo (SSA) and aerosol optical depth (AOD) were calculated using the following equations:

$\mathrm{SSA}\left( \lambda\right)= \frac{b_{scat}\left( \lambda\right)}{b_{scat}\left( \lambda\right)+b_{BC}\left( \lambda\right)+b_{BrC}\left( \lambda\right)}$, (S2)

$AOD=\sum_{i=1}^{n} Ext(\lambda)\tau_{i}=\sum_{i=1}^{n} {(b}_{scat}(\lambda)+b_{BC}(\lambda)+b_{BrC}(\lambda))\tau_{i}$, (S3)

where $Ext$ is the extinction coefficient averaged in the i-th altitude bin with a thickness of $\tau_{i}$. Vertically resolved data were binned every 3 km. An asymmetry parameter (g) of 0.65 [*Andrews et al.*, 2006] was used and the global direct radiative effect (DRE) was calculated under the condition of clear sky and a mean solar zenith angle computed from the specified date and geographic coordinates using an internal solar ephemeris algorithm. For a complete ATom deployment, the mean zenith angle used was ~60°. In all cases, the surface albedo was that of seawater since most flights were conducted over the ocean.

**S4. Calculation of Biomass Burning Potassium (K^+^_BB_)**

Water soluble potassium, (K^+^) has been widely used as a biomass burning tracer, however its application is complicated because of additional sources of K^+^, such as mineral dust and sea salt [*X Zhang et al.*, 2010]. Biomass burning K^+^ (K^+^_BB_) was calculated by K^+^_BB_=K^+^ -0.036*Na^+^ -0.12*(Ca^2+^_NSS_-Ca^2+^_BB_) [*Pio et al.*, 2008] to exclude K^+^ contributed from sea salt and mineral dust. All ions were measured by ion chromatography from filters extracts [*Dibb et al.*, 1999]. The factor of 0.036 is the ratio of K^+^ to Na^+^ in sea water and 0.12 is a mean crustal K^+^ to crustal Ca^2+^ mass ratio proposed by [*Pio et al.*, 2007]. Biomass burning Ca^2+^ (Ca^2+^_BB_) can be estimated by 10 times biomass burning K^+^ (K^+^_BB_), and non-sea-salt Ca^2+^ (Ca^2+^_NSS_) can be calculated from the difference between overall measured Ca^2+^ and Ca^2+^ related with sea salt [*Pio et al.*, 2008].


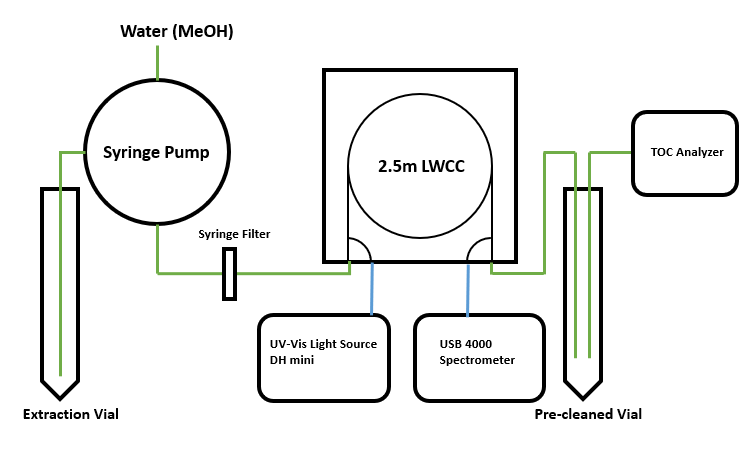


**Figure S1.** Schematic of the bench top laboratory instrument to measure aerosol Brown Carbon (BrC) and Water-Soluble Organic Carbon (WSOC) from filter extracts. The syringe filter is 0.45 μm pore size polypropylene (Tisch Scientific, North Bend, OH). An identical system was used for measuring BrC from the extraction of the filter in methanol, but without the TOC analysis (sample discarded after LWCC). WSOC data are not discussed in this paper.


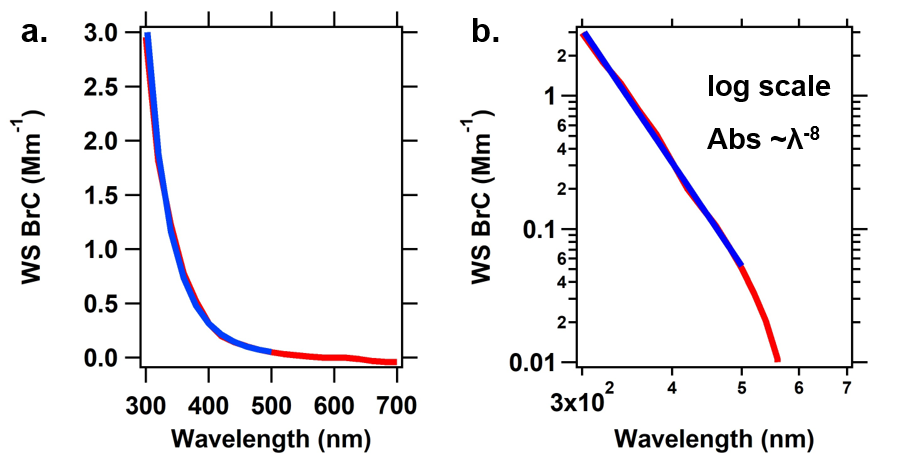


**Figure S2.** Example absorption spectrum (red) of filter extracts. Blue line is the fit line from 300 nm to 500 nm and resulting predicted Angstrom exponent of 8 based on linear regression of the log-transformed variables. Plot (a) is on a linear scale and (b) log-log scale.


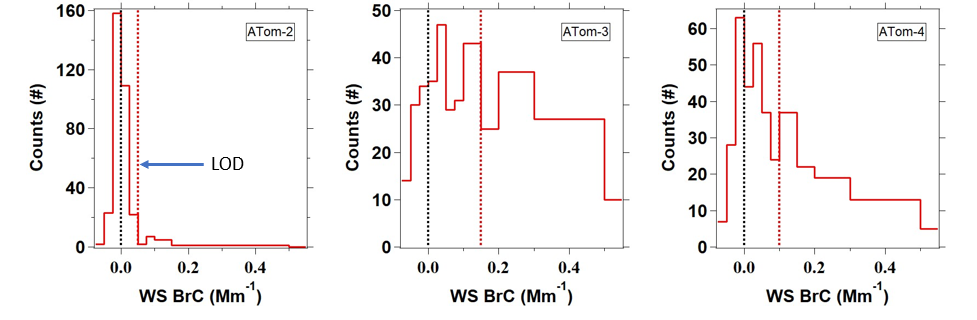


**Figure S3.** Frequency distribution of water-soluble BrC measurements (absorption coefficient at 365nm, see Eq(1) in main text) with filter blank correction. The vertical red dotted line is the estimated LOD for each mission based on three times the standard deviation of the blanks, and vertical black dotted line is the zero line. Negative values are when the ambient measurement is less than the filter blank.


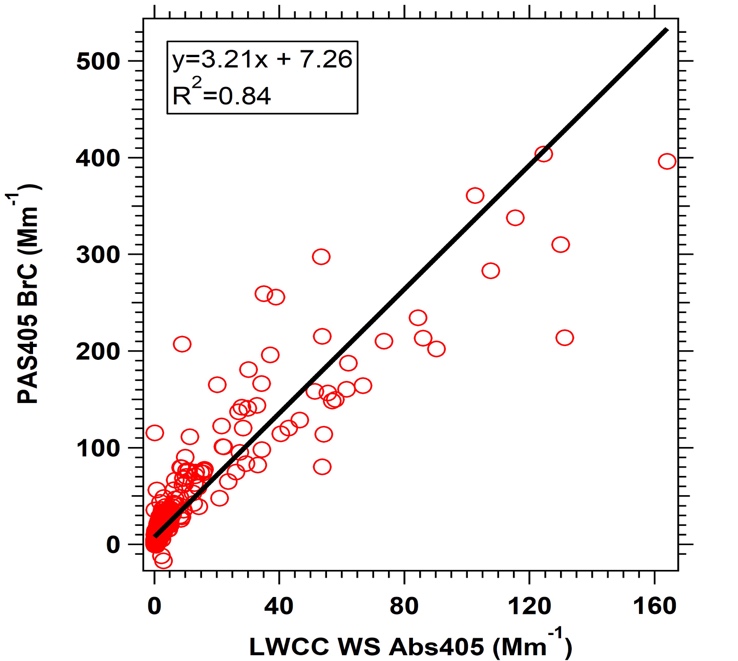


**Figure S4.** Comparison of aerosol total BrC light absorption coefficients measured at a wavelength of 405 nm by a photoacoustic aerosol absorption spectrometer (PAS405 BrC) and water-soluble BrC in solution measured with the filter/liquid wave guide capillary cell (LWCC WS BrC Abs405) photospectrometer deployed in this study. Data are from the recent NASA FIREX-AQ study of smoke plumes in the western USA utilizing the same aircraft and identical filter sampling system, and same BrC analysis and data processing as that used for ATom. The PAS lowest measurement wavelength was 405 nm, which was use in this direct comparison. BrC was determined from the PAS aerosol absorption data (PAS405 BrC) by assuming a BC AAE of 1, a MAC of 10 m^2^/g at 660 nm and using the measured SP2 BC mass, the same parameters used for determining the wavelength dependent BC absorption coefficients in the ATom radiative model. (This results in a BC MAC of 16.3 m^2^/g at 405 nm; from $10m^{2}/g\times\frac{660nm}{405nm}$). Then, PAS405 BrC = (PAS measured absorption at 405 nm) – (16.3 m^2^/g) (SP2 BC mass). WS-BrC was determined from spectrophotometer measurements at 405 nm and converted to absorption coefficient by Eq(S1). The slope in the plot, which is the conversion factor between WS BrC and actual BrC at 405 nm, was determined by orthogonal regression.


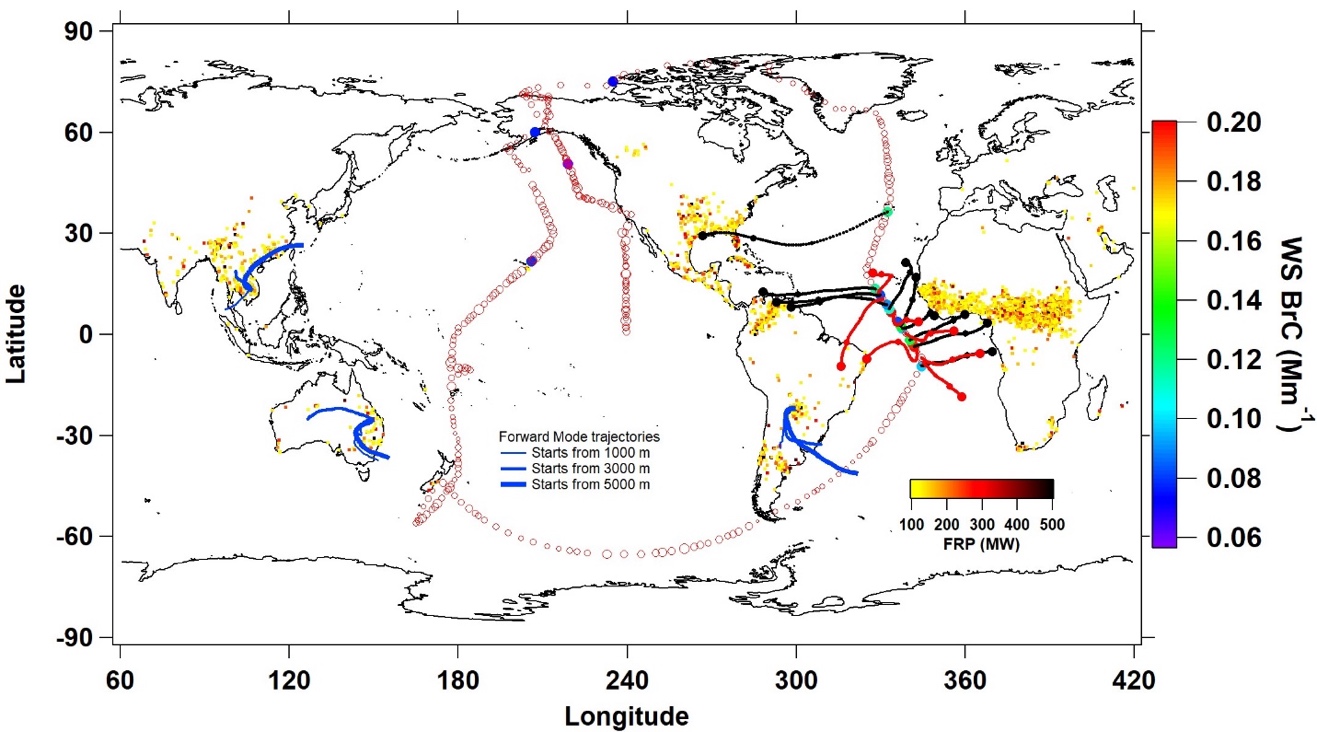


**Figure S5.** Forward trajectories (blue) at various altitudes for regions of burning at times when the plume could be intercepted by the DC8 aircraft during ATom-2. For example, the fires in Southeast Asia and Australia did not reach or were not transported in the direction of the aircraft during roughly the time the DC8 was in the southern Pacific Ocean. For the fires in southern Africa that passed over the aircraft sampling path within 3 days of emission, the altitude did not intersect with the aircraft sampling location.


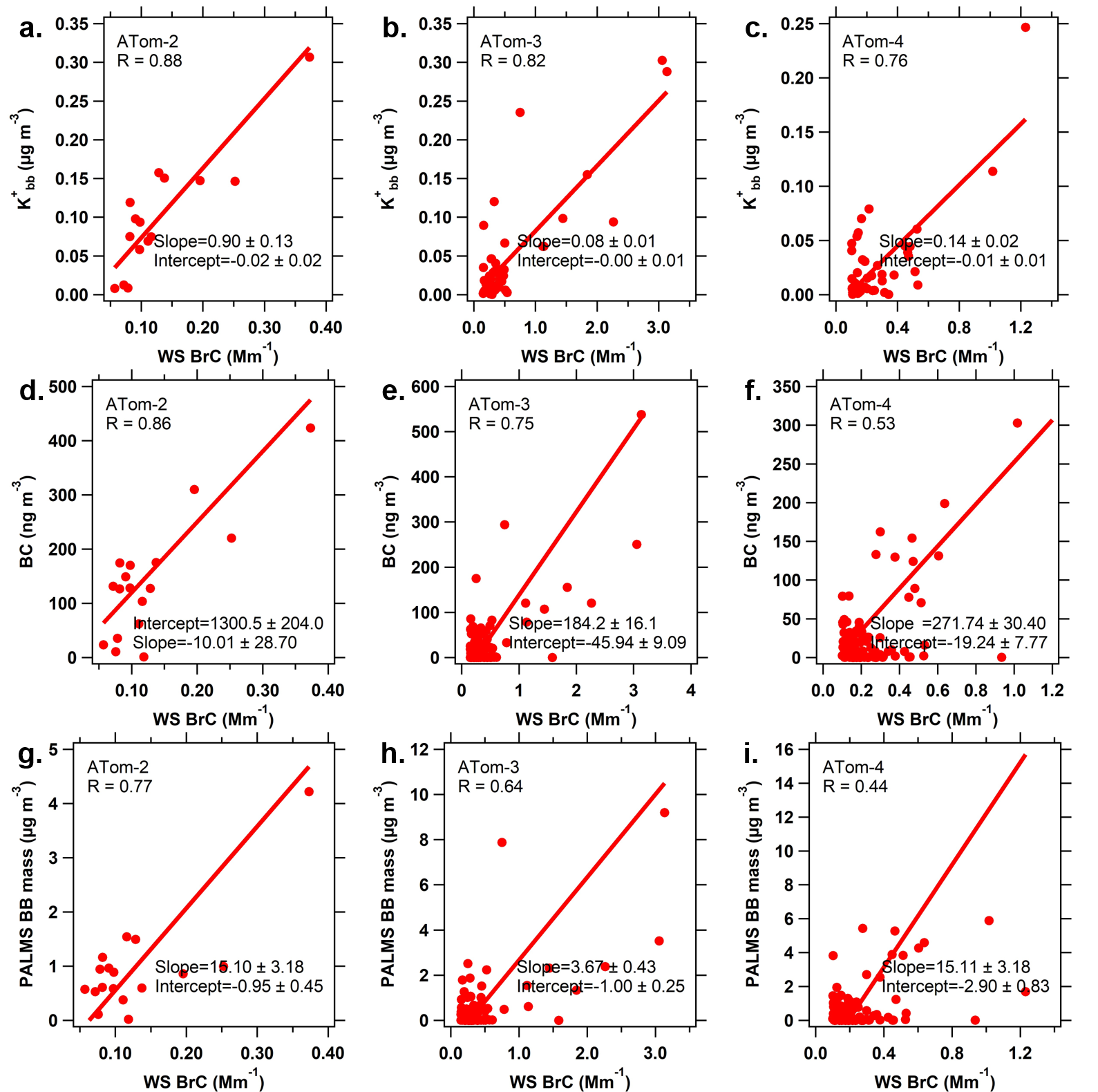


**Figure S6.** Relationship between WS BrC, biomass burning potassium (K^+^_BB_), BC, and PALMS tracer analysis of biomass burning aerosol mass. Only data above the LOD are included. All plots have a p-value less than 0.01. WS BrC is the absorption coefficient at 365 nm determined from the LWCC by Eq(1) in the main text.


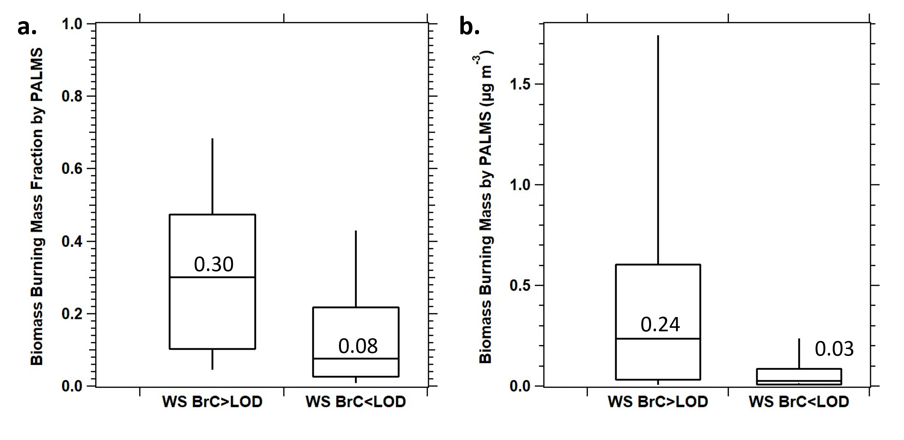


**Figure S7.** Summary of single particle tracer analysis of biomass burning contribution to aerosols 0.1 to 4.8 µm diameter for periods when WS BrC > LOD and and WS BrC < LOD; (a) biomass burning percent mass fraction, and (b) biomass burning mass concentration. Data are for combined ATom 2, 3 and 4 missions. Middle line in box is median, with the value given above the line, the lower and upper edges of the box are the lower (25%) and upper (75%) quartiles and the line extend to the lower and upper extremes (10 and 90%).


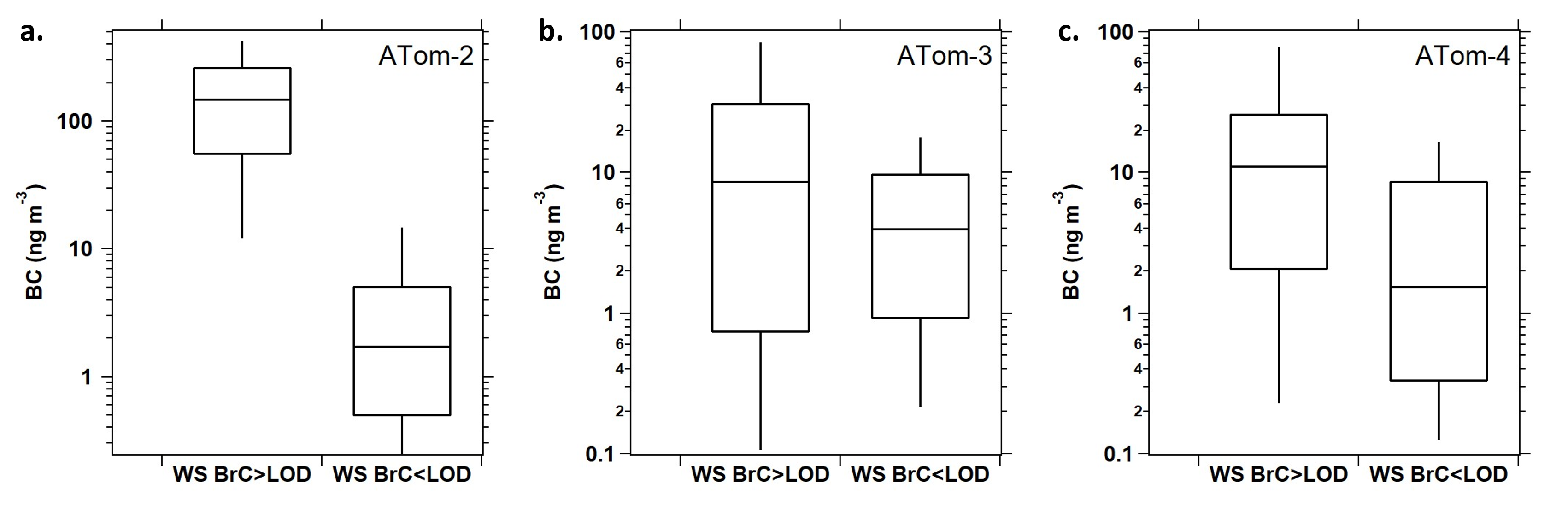


**Figure S8.** Comparison for each ATom mission of BC mass measured by the SP2 for data when BrC was greater than LOD and when BrC was less than the LOD. BC was always higher when BrC>LOD, and was substantially higher for ATom mission 2. The results are consistent with high levels of BC being associated with incomplete combustion, such as biomass burning, a known strong source for BrC. In the plots the middle line in the box is the median, the lower and upper edges of the box are the lower (25%) and upper (75%) quartiles and the line extend to the lower and upper extremes (10 and 90%).


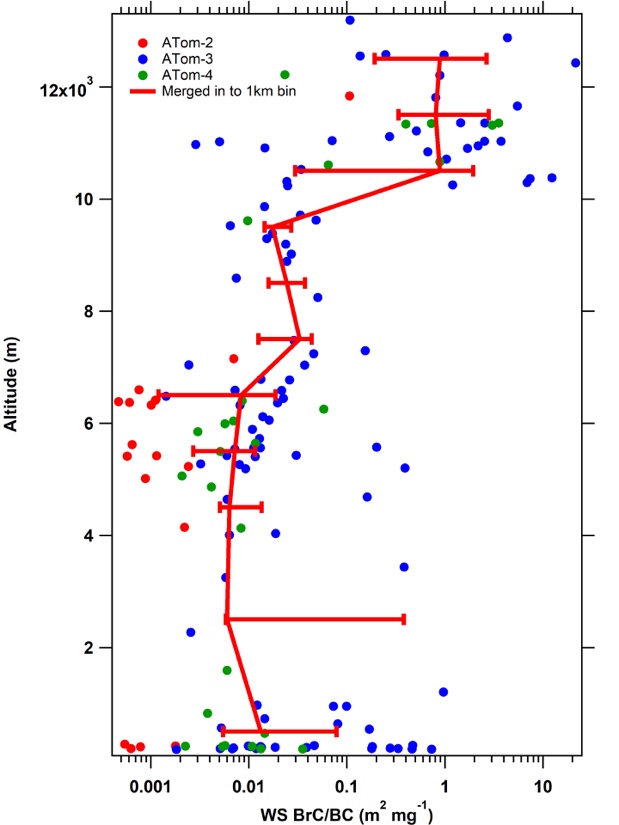


**Figure S9.** Vertical profiles of WS BrC to BC (ratio). Data are averaged over 1 km altitude bins. Data for 2.5 km are merged from 1 km to 4 km due to fewer data points. Error bars are the interquartile range of the ratio.

**Table S1.** Pearson correlations (r).

|  | ATom-2 | ATom-3 | ATom-4 |
| --- | --- | --- | --- |
| WS BrC and BC | 0.86 | 0.75 | 0.53 |
| WS BrC and K^+^_BB_ | 0.88 | 0.82 | 0.76 |
| WS BrC and PALMS BB mass | 0.77 | 0.64 | 0.44 |
| BC and K^+^_BB_ | 0.92 | 0.85 | 0.45 |

**Table S2.** Mean DRE due to absorption by BrC and BC, and dry particle scattering by longitude range for combined ATom-2, 3 and 4. Numbers in parentheses are results from using zero as the WS BrC data less than LOD.

|  |  | DRE due to absorption by BrC (W m^-2^) | DRE due to absorption by BC (W m^-2^) | Scattering (W m^-2^) | The fraction of total carbonaceous aerosol DRE by BrC |
| --- | --- | --- | --- | --- | --- |
| Arctic | ATom-2 | 0.013 (0.002) | 0.063 | -6.013 | 17% (3%) |
| 60N – 90N | ATom-3 | 0.040 (0.031) | 0.092 | -4.633 | 30% (25%) |
|  | ATom-4 | 0.085 (0.058) | 0.102 | -4.277 | 46% (36%) |
| N Subtropical | ATom-2 | 0.029 (0.005) | 0.126 | -6.184 | 19% (4%) |
| 20N – 60N | ATom-3 | 0.147 (0.097) | 0.064 | -11.675 | 70% (60%) |
|  | ATom-4 | 0.179 (0.138) | 0.371 | -13.166 | 33% (27%) |
| Tropical | ATom-2 | 0.051 (0.023) | 0.188 | -9.196 | 21% (11%) |
| 20S – 20N | ATom-3 | 1.204 (1.404) | 1.664 | -42.474 | 42% (46%) |
|  | ATom-4 | 0.185 (0.152) | 0.141 | -9.648 | 57% (52%) |
| S Subtropical | ATom-2 | 0.029 (0) | 0.017 | -10.680 | 63% (0) |
| 60S – 20S | ATom-3 | 0.151 (0.098) | 0.220 | -12.261 | 41% (31%) |
|  | ATom-4 | 0.103 (0.049) | 0.041 | -8.376 | 71% (54%) |
| Antarctic | ATom-2 | 0.012 (0) | 0.003 | -5.823 | 79% (0) |
| 90S – 60S | ATom-3 | 0.037 (0.040) | 0.030 | -3.217 | 56% (58%) |
|  | ATom-4 | 0.057 (0.023) | 0.005 | -3.681 | 93% (83%) |

*Z. Angle is the mean solar Zenith Angle used in the radiative transfer calculation.

**References:**

Andrews, E., et al. (2006), Comparison of methods for deriving aerosol asymmetry parameter, *Journal of Geophysical Research: Atmospheres*, *111*(D5), doi:10.1029/2004jd005734.

Dibb, J. E., R. W. Talbot, E. M. Scheuer, D. R. Blake, N. J. Blake, G. L. Gregory, G. W. Sachse, and D. C. Thornton (1999), Aerosol chemical composition and distribution during the Pacific Exploratory Mission (PEM) Tropics, *Journal of Geophysical Research: Atmospheres*, *104*(D5), 5785-5800, doi:10.1029/1998jd100001.

Hecobian, A., X. Zhang, M. Zheng, N. Frank, E. S. Edgerton, and R. J. Weber (2010), Water-Soluble Organic Aerosol material and the light-absorption characteristics of aqueous extracts measured over the Southeastern United States, *Atmos. Chem. Phys.*, *10*(13), 5965-5977, doi:10.5194/acp-10-5965-2010.

Liu, J., M. Bergin, H. Guo, L. King, N. Kotra, E. Edgerton, and R. J. Weber (2013), Size-resolved measurements of brown carbon in water and methanol extracts and estimates of their contribution to ambient fine-particle light absorption, *Atmos. Chem. Phys.*, *13*(24), 12389-12404, doi:10.5194/acp-13-12389-2013.

McNaughton, C. S., et al. (2007), Results from the DC-8 Inlet Characterization Experiment (DICE): Airborne Versus Surface Sampling of Mineral Dust and Sea Salt Aerosols, *Aerosol Science and Technology*, *41*(2), 136-159, doi:10.1080/02786820601118406.

Pio, C. A., M. Legrand, C. A. Alves, T. Oliveira, J. Afonso, A. Caseiro, H. Puxbaum, A. Sanchez-Ochoa, and A. Gelencsér (2008), Chemical composition of atmospheric aerosols during the 2003 summer intense forest fire period, *Atmospheric Environment*, *42*(32), 7530-7543, doi:<https://doi.org/10.1016/j.atmosenv.2008.05.032>.

Pio, C. A., et al. (2007), Climatology of aerosol composition (organic versus inorganic) at nonurban sites on a west-east transect across Europe, *Journal of Geophysical Research: Atmospheres*, *112*(D23), doi:10.1029/2006JD008038.

Wofsy, S. C., et al. (2018), ATom: Merged Atmospheric Chemistry, Trace Gases, and Aerosols, edited, ORNL Distributed Active Archive Center, doi:10.3334/ornldaac/1581.

Wooster, M. J., G. Roberts, G. L. W. Perry, and Y. J. Kaufman (2005), Retrieval of biomass combustion rates and totals from fire radiative power observations: FRP derivation and calibration relationships between biomass consumption and fire radiative energy release, *Journal of Geophysical Research: Atmospheres*, *110*(D24), doi:10.1029/2005JD006318.

Zhang, X., A. Hecobian, M. Zheng, N. H. Frank, and R. J. Weber (2010), Biomass burning impact on PM<sub> 2.5</sub> over the southeastern US during 2007: integrating chemically speciated FRM filter measurements, MODIS fire counts and PMF analysis, *Atmos. Chem. Phys.*, *10*(14), 6839-6853, doi:10.5194/acp-10-6839-2010.

Zhang, Y., et al. (2017), Top-of-atmosphere radiative forcing affected by brown carbon in the upper troposphere, *Nature Geoscience*, *10*, 486, doi:10.1038/ngeo2960

<https://www.nature.com/articles/ngeo2960#supplementary-information>.
